# Interplay among RNA polymerases II, IV and V in RNA-directed DNA methylation at a low copy transgene locus in *Arabidopsis thaliana*

**DOI:** 10.1007/s11103-013-0041-4

**Published:** 2013-03-20

**Authors:** Wanhui You, Zdravko J. Lorkovic, Antonius J. M. Matzke, Marjori Matzke

**Affiliations:** 1 Gregor Mendel Institute of Molecular Plant Biology, Austrian Academy of Sciences, 1030 Vienna, Austria; 2 Department of Molecular Biology, Faculty of Science, University of Zagreb, Horvatovac 102a, Zagreb, Croatia; 3 Institute of Plant and Microbial Biology, Academia Sinica, 128, Sec. 2, Academia Rd., Nankang, Taipei, 115 Taiwan

**Keywords:** Non-coding RNA, RNA polymerase II, Pol IV, Pol V, RNA-directed DNA methylation, Secondary siRNA, siRNA amplification

## Abstract

RNA-directed DNA methylation (RdDM) is an epigenetic process whereby small interfering RNAs (siRNAs) guide cytosine methylation of homologous DNA sequences. RdDM requires two specialized RNA polymerases: Pol IV transcribes the siRNA precursor whereas Pol V generates scaffold RNAs that interact with siRNAs and attract the methylation machinery. Recent evidence also suggests the involvement of RNA polymerase II (Pol II) in recruiting Pol IV and Pol V to low copy, intergenic loci. We demonstrated previously that Pol V-mediated methylation at a transgene locus in *Arabidopsis* spreads downstream of the originally targeted region by means of Pol IV/RNA-DEPENDENT RNA POLYMERASE2 (RDR2)-dependent 24-nt secondary siRNAs. Here we show that these secondary siRNAs can not only induce methylation in *cis* but also in *trans* at an unlinked target site, provided this sequence is transcribed by Pol II to produce a non-coding RNA. The Pol II transcript appears to be important for amplification of siRNAs at the unlinked target site because its presence correlates not only with methylation but also with elevated levels of 24-nt siRNAs. Potential target sites that lack an overlapping Pol II transcript and remain unmethylated in the presence of *trans*-acting 24-nt siRNAs can nevertheless acquire methylation in the presence of 21–24-nt hairpin-derived siRNAs, suggesting that RdDM of non-transcribed target sequences requires multiple size classes of siRNA. Our findings demonstrate that Pol II transcripts are not always needed for RdDM at low copy loci but they may intensify RdDM by facilitating amplification of Pol IV-dependent siRNAs at the DNA target site.

**Electronic supplementary material:**

The online version of this article (doi:10.1007/s11103-013-0041-4) contains supplementary material, which is available to authorized users.

## Introduction

RNA-directed DNA methylation (RdDM) is a small interfering (si) RNA-mediated epigenetic modification that contributes to transcriptional gene silencing (TGS) of transposons and repetitive sequences in plants. RdDM requires an intricate transcriptional machinery that centers around two plant-specific, RNA polymerase II (Pol II)-related enzymes called Pol IV and Pol V (Haag and Pikaard 2011). In the canonical RdDM pathway, Pol IV is responsible for producing or amplifying the siRNA trigger whereas Pol V is thought to synthesize a scaffold RNA that interacts with siRNAs and recruits the methylation machinery to the DNA target site (He et al. 2011; Wierzbicki 2012; Eun et al. 2012). Characteristic features of RdDM include methylation of cytosines in all sequence contexts (CG, CHG and CHH, where H is A, T or C) and restriction of methylation to the region of siRNA-DNA sequence homology.

Although Pol IV and Pol V have received the most attention in studies of RdDM, a recent investigation has revealed a role for Pol II in coordinating the activities of Pol IV and Pol V at intergenic, low copy number (Type II) loci. Using a weak allele in the gene encoding NRPB2, the second largest subunit of Pol II, Zheng and coworkers showed that Pol II is able to recruit both Pol IV and Pol V to chromatin at Type II loci, thereby coordinating their functions in siRNA accumulation and TGS, respectively (Zheng et al. 2009).

Here we describe a transgene silencing system in *Arabidopsis thaliana* (*Arabidopsis*) that illustrates further the contribution of Pol II to the RdDM pathway. Our findings were made during experiments designed to test whether Pol IV/RNA-DEPENDENT RNA POLYMERASE2 (RDR2)-dependent, 24-nt secondary siRNAs, which induce methylation in *cis* at the site where they are generated (Daxinger et al. 2009), can also act in *trans* to elicit methylation of an unlinked homologous target sequence. Consistent with a role for Pol II in RdDM at low copy target loci, the secondary siRNAs were able to trigger methylation in *trans* but only at target sequences that are transcribed by Pol II to produce an overlapping non-coding RNA.

## Materials and methods

### Plant materials

**Fig. 1:**
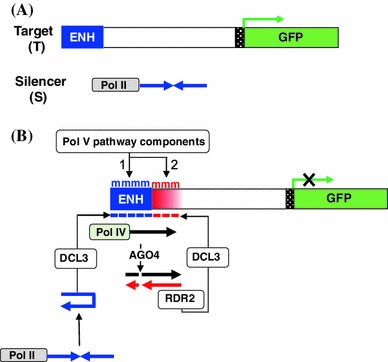
T + S transgene silencing system and model for production of secondary siRNAs. **a** The two-component transgene silencing system comprises a Target locus (*T*) and a Silencer locus (*S*). The T locus contains a *GFP* reporter gene under the control of a minimal promoter (*hatched*) and an enhancer (ENH) that drives *GFP* expression in shoot and root meristem regions. The S locus contains an inverted DNA repeat (*IR*) of target enhancer sequences (opposing *blue arrows*) that is transcribed from the cauliflower mosaic virus 35S promoter by Pol II (Kanno et al. 2008). **b** The resulting RNA hairpin from the S locus is processed by DICER-LIKE3 (DCL3) into 24-nt primary siRNAs (*blue dashes*) that induce Pol V-mediated methylation of the target enhancer (*blue* ‘m’) leading to TGS of the *GFP* reporter gene (step 1). An additional feature of this system (step 2) is that methylation spreads (*red* ‘m’) from the originally targeted enhancer into the downstream region (*red shaded bar*) through the presence of secondary siRNAs (*red dashes*), which rely on Pol IV and RDR2 for their biogenesis (Daxinger et al. 2009). Key to secondary siRNA production is a ‘nascent’ RNA that extends through the target enhancer region (*black arrow*). In a hypothetical model, the nascent RNA is transcribed by Pol IV and following cleavage by ARGONAUTE 4 (AGO4), is copied by RDR2 into double stranded RNA that is processed by DCL3 into 24-nt secondary siRNAs (Daxinger et al. 2009)

All experiments were performed using *Arabidopsis thaliana* accession *Col*-0. Transgenic plants containing only the target (T) locus or the T locus and silencer (S) locus (Fig. 1) were used as described previously (Kanno et al. 2008; Daxinger et al. 2009; Lorković et al. 2012; Eun et al. 2012). For the mutants defective in the largest subunits of Pol IV and Pol V, respectively, the following alleles were used: *nrpd1*-*7* (Smith et al. 2007) and *nrpe1*-*3* (Kanno et al. 2010). Primers for genotyping are shown in Supplementary Table 1. Plants were grown under a 16 h light/8 h dark cycle at ~23 °C in either a greenhouse or growth chamber. A list of plants used for analysis is shown in Table 1.

**Table 1:** List of plants used in this study and summary of results

Line	Methylation of 88 bp target sequence (SD locus)	Non-coding Pol II transcript (SD locus)	Pol IV-dependent *trans*-acting secondary siRNAs (T + S)	Amplified siRNAs (SD locus)
T	n.a.	n.a.	No	n.a.
T + S	n.a.	n.a.	Yes	n.a.
T + SD #1 to #6	No	n.d.	n.a.	n.a.
T + S + SD #1	Yes	Yes	Yes	Yes
T + S + SD #2	No	No	Yes	No
T + S + SD #3	No	No	Yes	No
T + S + SD #4^c^	Yes	Yes	Yes	Yes
T + S + SD#1 *nrpd1*	No	n.d	No^a^	n.d.
T + S + SD#1 *nrpe1*	No	n.d.	No^a^	n.d.
T + S + SD#4 *nrpd1* ^b,c^	No	Yes	No^a^	No
T + S + SD#4 *nrpe1*	No	Yes	No^a^	n.d.
SD(#4)^b,c^	Reduced	Yes	No	No
SD(#2) + 88-bp HP	Yes	No	No (HP-derived siRNAs)	No

*n.a.* not applicable, *n.d.* not determined

^a^Pol IV/RDR2-dependent secondary siRNAs are not made in the T + S system in *nrpd1* and *nrpe1* mutant backgrounds (Daxinger et al. (2009))

^b^These lines demonstrate that the Pol II transcript is unable to support full methylation in the absence of 24-nt siRNAs and that siRNA amplification does not occur in plants that contain the Pol II transcript but not the *trans*-acting secondary siRNAs

^c^These lines illustrate the proposed indirect role of Pol II in siRNA amplification because the Pol II transcript accumulates to similar levels whether siRNAs are amplified (T + S + SD #4) or not [T + S + SD #4 *nrpd1* mutant or SD(#4)]

### Plasmid constructs

**Fig. 2:**
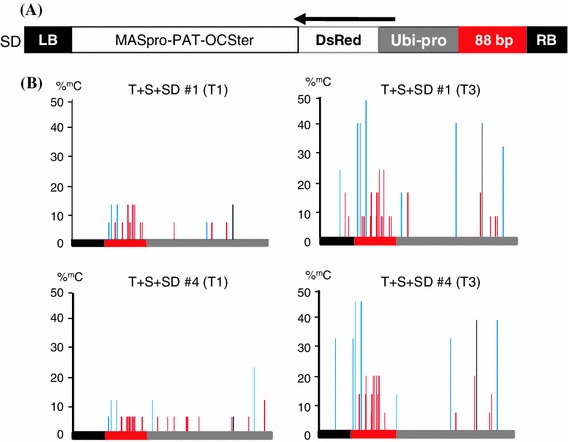
SD construct and methylation analysis of 88 bp target region. **a** The SD construct contains the *88* *bp*-*Ubi*-*pro*-*DsRed* sequence (Supplementary Fig. 1) in the orientation shown relative to the T-DNA *left* and *right borders* (LB and RB, respectively) together with a *PAT* (phosphinothricin acetyl transferase) gene encoding resistance to phosphinothricin under the control of the mannopine synthase promoter (MASpro) and octopine synthase terminator (OCSter) for selection of transformed plant cells (construct not drawn to scale). The *black arrow* indicates the predicted *DsRed* transcript initiating from the Ubi-pro. **b** Bisulfite sequencing analysis of methylation in the 88 bp target sequence (*red bar*) and immediate flanking sequences (*left black bar* represents vector sequence; *right grey bar* represents Ubi-pro sequence). Note that these fragments are shown in 5′–3′ orientation, which is reverse to that shown in Part A. The 88 bp target sequence contains 19 cytosines: four in a CHG context (*blue lines*), 15 in a CHH context (*red lines*) and none in a CG context (*black lines*) (Supplementary Fig. 1). Percent cytosine methylation is shown for T + S + SD lines #1 and #4. Methylation was first observed in the T1 generation (*left*), in which the SD locus is hemizygous, and it persisted and even increased by the T3 generation (*right*) when the SD locus is homozygous. In T + S + SD lines #2 and #3, no methylation was detected. In T + S + SD line #5, sparse methylation was observed in T1 plants, but CHH methylation did not persist into the T3 generation (data not shown). The results from at least 15 cloned sequences are shown

The ‘SD’ (siRNA-DNA) construct (Fig. 2a, Supplementary Fig. 1) contains an 88 bp target sequence to be tested for acquisition of methylation in the presence of potentially *trans*-acting secondary siRNAs. In the T + S silencing system, the 88 bp sequence is directly downstream of the enhancer targeted for methyation by hairpin-derived primary siRNAs and it corresponds to the major region acquiring methylation through Pol IV-dependent, *cis*-acting secondary siRNAs (Daxinger et al. 2009) (Fig. 1b). For the SD construct used in the studies reported here, the 88 bp sequence was positioned upstream of a maize ubiquitin promoter (Ubi-pro) (Christensen et al. 1992; Christensen and Quail 1996) driving expression of a gene encoding red fluorescent protein (DsRed) (Fig. 2a, Supplementary Fig. 1). The 88 bp-Ubi-pro-DsRed fragment was inserted into the MPO (Mannopine promoter, Phosphinothricin and Octopine terminator) binary vector (Matzke et al. 2010).

The 88 bp HP (hairpin) construct was designed to contain an inverted DNA repeat (IR) of the 88 bp sequence, with the two halves separated by the α′ promoter sequence (Kanno et al. 2008), under the control of the 35S promoter (35Spro) of cauliflower mosaic virus. The 35Spro-88 bp-IR fragment was synthesized by Mr. Gene (Regensburg, Germany) and inserted into a binary vector of the pPZP 200 series (Hajdukiewicz et al. 1994).

The two engineered binary vectors were introduced into *Agrobacterium tumefaciens* using triparental mating (Matzke and Matzke 1986). Transgenic *Arabidopsis* plants were obtained by using floral dip method (Clough and Bent 1998). The T1 generation corresponds to plants grown from seeds of transformed plants. Subsequent selfed generations are referred to as T2, T3 and so on.

### Bisulfite sequencing analysis

Genomic DNA was isolated from rosette leaves of T1 plants or T3 seedlings using a DNeasy Plant Mini kit (Qiagen). Before bisulfite conversion, 1.5 micrograms of genomic DNA was digested with *Hin*dIII, which cannot cut the target fragment. The digested DNA was purified using a QIAquick PCR Purification Kit (Qiagen). Bisulfite treatment of the purified DNA was carried out using an EpiTect Bisulfite Kit (Qiagen) according to the manufacturer’s instructions with the following modifications: the conversion PCR programme was changed into 95 °C 2 min, 75 °C 2 h, 95 °C 1 min for 9 cycles, and hold at 75 °C. The target fragment PCR reactions were performed using Advantage 2 Polymerase Mix (Clontech) and the conditions for the amplification of bisulfite-treated DNA were as follows: 95 °C for 5 min followed by 39 cycles at 95 °C for 30 s, 30 s annealing temperature for a particular primer pair, 72 °C for 1 min, and 5 min of final elongation. PCR was carried out in a total reaction volume of 50 μl. The PCR product was gel-purified with QIAquick Gel Extraction Kit (Qiagen), ligated into pGEM-T Easy Vector (Promega), and followed by a normal transformation procedure with white-blue selection. Colony PCR was performed with M13 primers, using the selected white colonies as templates. PCR products were sent for sequencing after purification with ExoSAP-IT (Affymetrix). At least 15 clones were used for bisulfite sequencing analysis. Colony PCR conditions: 95 °C 10 min, 95 °C 30 s, 55 °C 30 s, 72 °C 1 min for 40 cycles, 72 °C 5 min in a total 10 μl final volume with M13 primers. ExoSAP-IT treatment 1:100 dilution from original solution and pipet 2 μl into 10 μl Colony PCR product, incubate at 37 °C overnight, inactivate at 80 °C 15 min, store at 4 °C until further use. As a control for complete bisulfite conversion, we used the PHAVOLUTA gene: PCR conditions are the same as above except for the first pair of primers we used 40 cycles and for the second pair 26 cycles. Primers used are listed in Supplementary Table 1.

### Small RNA isolation and Northern blot analysis

Small RNAs were isolated from mixed inflorescence tissues pooled from several plants using the mirVana miRNA isolation kit (Ambion/Applied Biosystems) and analyzed by Northern blot hybridization as described previously (Kanno et al. 2005; Huettel et al. 2006; Daxinger et al. 2009). To detect siRNAs originating from the 88 bp sequence (Supplementary Fig. 1), the following end-labeled oligonucleotide probe was used: TTC GAT TAT GAA TAA TAA ACA GGC TGC ATC TTC AGG CAT CC.

### Non-coding RNA analysis

To detect transcripts from the SD construct, total RNA was extracted from 3 week-old seedlings (total wet weight approximately 100 mg) by using TRIzol^®^ Reagent (Invitrogen). Approximately 1 microgram of total RNA was used for reverse transcription using RevertAid™ H Minus First Strand cDNA Synthesis Kit (Fermantas) according to the manufacturer’s instructions. After this step, 1 μl of cDNA was used for semi-quantitative reverse transcriptase–mediated (RT) PCR analysis. The PCR conditions were 95 °C for 5 min followed by 23 (*ACTIN*) or 40 (non-coding RNA) amplification cycles (95 °C for 30 s, 55 °C for 30 s, and 72 °C for 1 min). Actin was used as an internal control. Primers used in semi-quantitative RT-PCR are listed in Supplementary Table 1 and those relevant to the SD construct sequence are shown in context in Supplementary Fig. 1.

### Chromatin immunoprecipitation assay

Chromatin immunoprecipitation was performed as described in http://mescaline.igh.cnrs.fr/EpiGeneSys/images/stories/protocols/pdf/20111025150640_p13.pdf. The chromatin was immunoprecipitated with antibody against Pol II (Millipore). Real-time PCR analysis was performed with a Bio-Rad iQ5 machine using SensiFAST mix (Bioline). All data are expressed relative to input. The results shown were reproduced in two biological replicates. The primer sets used for the Real-time PCR are listed in Supplementary Table 1 and their positions within the SD construct in Supplementary Fig. 1. These primers were chosen because they are specific for the SD construct and they produced single PCR amplification products.

## Results

In the T + S transgene silencing system, Pol IV/RDR2-dependent, 24-nt secondary siRNAs are involved in spreading of methylation approximately 100 bp downstream of a target enhancer sequence, which itself acquires methylation in the presence of hairpin-derived primary siRNAs that are 21–24-nt in length (Fig. 1a, b). The proposed model for secondary siRNA biogenesis involves synthesis and turnover of a Pol IV-generated ‘nascent’ RNA that extends from the target enhancer into the downstream region (Fig. 1b) (Daxinger et al. 2009). While the Pol IV/RDR2-dependent secondary siRNAs are able to induce methylation in *cis* at the site where they are produced, an open question is whether they would also be able to trigger methylation in *trans* at unlinked homologous target sites.

To investigate this question, a transgene construct (‘SD’) bearing a new target sequence comprising 88 bp from the region directly downstream of the original target enhancer was assembled. The 88 bp region is included in the approximately 100 bp segment that is methylated by *cis*-acting secondary siRNAs. In the SD construct, the 88 bp target sequence is positioned upstream of a maize ubiquitin promoter (Ubi-pro) that drives expression of a gene encoding red fluorescent protein (DsRed) (Fig. 2a; Supplementary Fig. 1). The 88 bp sequence could thus be used to test the ability of Pol IV-dependent, 24-nt secondary siRNAs to elicit *trans*-RdDM of a low copy, non-protein-coding target DNA sequence.

The SD construct was introduced into the doubly homozygous T + S line using *Agrobacterium*-mediated transformation. As a control to test the dependence of any observed methylation on *trans*-acting 24-nt secondary siRNAs, the SD construct was also introduced into the original T line, which lacks secondary siRNAs owing to the absence of the S locus that is needed to initiate secondary siRNA biogenesis (Fig. 1b) (Daxinger et al. 2009). New triply transformed (T + S + SD) and doubly transformed (T + SD) lines were screened for single locus insertions of the SD construct by scoring for a 3 to 1 segregation of a linked antibiotic resistance marker in second generation (T2) seedlings. Five independent T + S + SD lines and six independent T + SD lines were retained for further analysis.

Bisulfite sequencing was used to analyze DNA methylation at the 88 bp target sequence in the T + S + SD and T + SD lines. Persistent methylation of cytosines in CHG and CHH trinucleotides was detected at the 88 bp target sequence in only two of the five T + S + SD lines (#1 and #4) (Fig. 2b). Despite some spreading into the immediate upstream and downstream sequences, this methylation was largely concentrated in the 88 bp target region. The relatively strict targeting of methylation and the presence of CHH methylation are features consistent with RdDM. In the T + SD lines, no methylation of the 88 bp target sequence was detected (data not shown). This result supports the idea that the methylation observed in the T + S + SD lines #1 and #4 was due to the Pol IV-dependent 24-nt secondary siRNAs acting in *trans*.

**Fig. 3:**
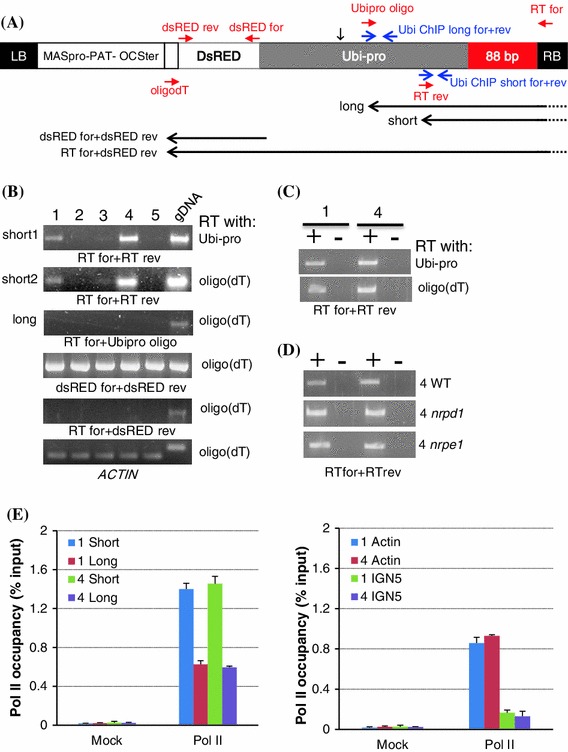
RT-PCR analysis of Pol II transcripts and Pol II occupancy. **a** Positions of primers used for RT-PCR analysis to detect transcripts from the *88* *bp*-*Ubi*-*pro*-*DsRed* sequence (short *red arrows*) and to study Pol II occupancy (short *blue arrows*) (see also Supplementary Fig. 1). *Longer black arrows* indicate transcripts potentially detected in RT-PCR reactions. Only the ‘short’ and dsREDfor + dsREDrev transcripts were detected (Part B). The *short black vertical arrow* indicates the approximate position of the predicted transcription start site of the *DsRed* transcript, which is around 150 bp downstream of the Ubipro oligo (Supplementary Fig. 1). **b** Reverse transcriptase (*RT*) reactions to synthesize first strand cDNA were carried out using either an Ubi-pro primer or oligo(dT) (*right* of each gel image). The primers used for the PCR reaction are shown under each gel image. The T + S + SD lines (#1 through #5) are labeled at the *top*. The ‘short’ transcript (A) overlapping the 88 bp region and extending into the Ubi-pro was observed only in lines #1 and #4 using either the Ubi-pro or oligo(dT) primer for RT and RTfor + RTrev primers for PCR (see minus RT controls in part C). The absence of the ‘long’ transcript [oligo(dT) in RT reaction and RTfor + Ubi-pro oligo for PCR] indicates that the non-coding transcript ends somewhere after the Ubi-pro oligo. In the RT reaction, the oligo(dT) is probably priming at A stretches upstream of the Ubi-pro oligo (Supplementary Fig. 1). A *DsRed* transcript (primers dsREDfor + dsREDrev) was observed in all five lines. However, expression of DsRed protein was only very weak or not detectable in these plants (data not shown). gDNA, genomic DNA. *ACTIN* was used as a constitutive control. **c** The ‘short1’ and ‘short2’ transcripts in lines T + S + SD #1 and #4 (part B) are not detected in minus RT (‘−’ sign) controls. ‘+’ sign indicates reactions with RT. **D.** The ‘short’ non-coding RNA overlapping the 88 bp target sequence was detectable in wild-type (*WT*) plants of T + S + SD line #4 but not in *nrpd1* or *nrpe1* mutant backgrounds. Two plants of each genotype were tested. The Ubi-pro primer was used for the RT reaction and the primer pair RTfor + RTrev for PCR. Plus and minus signs at the *top* indicate reactions with and without RT, respectively. **e** Pol II occupancy in the vicinity of the 88 bp target sequence. ChIP was performed using anti-Pol II antibody, and Pol II co-purified DNA was quantified by real-time PCR. Positions of primers used for the short (Ubi ChIP short for + rev) and long (Ubi ChIP long for + rev) fragments (*top graph*) are shown in Part A. Positive and negative controls for Pol II occupancy are Actin and IGN5 (*bottom graph*). Mock precipitations without antibody were used to judge background levels of ChIP samples. Two biological replicates were performed and SD were calculated from three technical repeats

Scaffold transcripts have been implicated in siRNA-mediated heterochromatin formation in fission yeast (Volpe et al. 2002) and RdDM in plants (Wierzbicki et al. 2008). Therefore, RT-PCR was used to test whether the presence of such transcripts could account for the differential methylation of the 88 bp sequence in the five T + S + SD lines. In these experiments, cDNA synthesis was primed with either an oligo(dT) or sequence-specific Ubi-pro primer and PCR amplification was then carried out using primer pairs distributed throughout the *88* *bp*-*Ubi*-*pro*-*DsRed* sequence (Fig. 3a, Supplementary Fig. 1). Consistent with the involvement of a scaffold transcript in methylation of the 88 bp target sequence, transcripts overlapping this sequence and part of the Ubi-pro were detected in the methylated T + S + SD lines #1 and #4 but not in the unmethylated lines #2, #3 and #5 (Fig. 3a, b, short1 and 2; minus RT controls in Fig. 3c). The transcripts overlapping the 88 bp region appeared to terminate within the Ubi-pro, probably somewhere just downstream of the Ubi-pro primer, because no PCR amplification product was observed when using oligo(dT) for cDNA synthesis and the primer combination RTfor + Ubi-pro for the PCR reaction (Fig. 3a, b, long). Although a *DsRed* transcript could be detected using oligo(dT)-primed cDNA synthesis and primers flanking the *DsRed* coding region for PCR amplification in all five T + S + SD lines (Fig. 3a, b, dsREDfor + dsREDrev), longer transcripts extending through the 88 bp region and continuing into the *DsRed* coding region were not observed (Fig. 3a, b, RTfor + dsREDrev). Collectively, the RT-PCR data suggest that the methylated T + S + SD lines #1 and #4 contain a non-coding RNA that overlaps the 88 bp target region and terminates in the Ubi-pro region, probably before the transcription start site for this promoter (Supplementary Fig. 1). These results thus provide a correlation between methylation of the 88 bp target sequence and non-coding transcripts overlapping this region.

Scaffold transcripts important for siRNA-mediated epigenetic modifications are produced by Pol II in fission yeast (Kato et al. 2005) and both Pol II and Pol V in plants (Zheng et al. 2009; Wierzbicki et al. 2008). It was thus of interest to identify the RNA polymerase responsible for synthesizing the non-coding transcripts overlapping the 88 bp region in the methylated T + S + SD lines. To test the involvement of Pol IV and Pol V, mutations in genes encoding the largest subunits of Pol IV and Pol V (*nrpd1* and *nrpe1*, respectively) were introduced into the T + S + SD line #4 and RT-PCR was used as before (Fig. 3a, b) to detect the ‘short’ transcript containing the 88 bp sequence. This transcript was still detectable in the *nrpd1* and *nrpe1* mutants (Fig. 3d), thus eliminating the possibility that either Pol IV or Pol V is involved in synthesizing the non-coding transcript and implicating instead Pol II. Because the 88 b target region is not predicted to be part of the *Ubi*-*pro*-*DsRed* transcription unit (Fig. 2a), the noncoding Pol II transcript overlapping this region would presumably initiate in a plant promoter in flanking plant DNA.

Attempts to directly test Pol II involvement by introgressing the *nrpb2*-*3* mutation (Zheng et al. 2009) into T + S + SD line #4 were not successful because the appropriate crosses did not yield viable progeny. In addition, this experiment is problematic in our system because Pol II-defective mutants would be impaired in production of the hairpin RNA encoded at the original S locus (Fig. 1b) and hence disrupt the entire silencing and RdDM cascade. However, the contribution of Pol II to synthesis of the non-coding RNA was substantiated by using chromatin immunoprecipitation (ChIP) to assess Pol II occupancy in the vicinity of the 88 bp target region. This analysis showed that Pol II occupancy was low around the Ubi-pro primer (Fig. 3a, e, Ubi ChIP longfor + rev) and higher at the upstream region that is closer to the 88 bp target sequence (Fig. 3a, e, Ubi ChIP short for + rev). These results support further the existence of a non-coding Pol II transcript initiating from an unidentified upstream plant promoter and extending through the 88 bp sequence to terminate within the Ubi-pro (Fig. 3a, ‘short’, Supplementary Fig. 1).

**Fig. 4:**
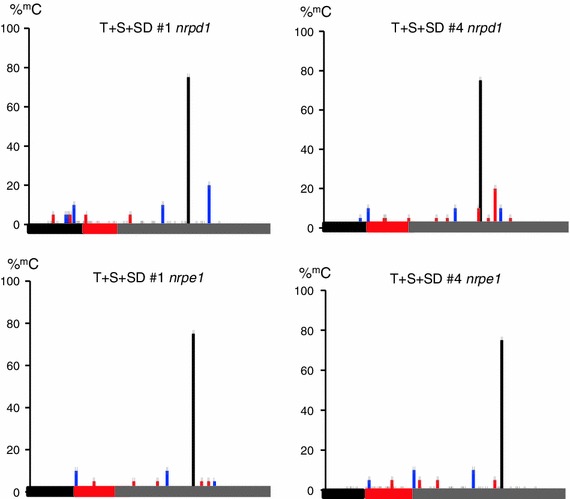
Methylation of the 88 bp target region in *nrpd1* and *nrpe1* mutants. Percent cytosine methylation in the 88 bp target sequence (*red bar*) and immediate flanking sequences (*left black bar*, vector sequence; *right grey bar*, Ubi-pro sequence) in lines T + S + SD #1 (left) and #4 (*right*) in *nrpd1* (*top*) and *nrpe1* (*bottom*) mutant backgrounds as determined by bisulfite sequencing. Methylation in CHH (*red lines*) and CHG (*blue lines*) nucleotide groups is substantially reduced relative to wild-type levels (see Fig. 2b). Major methylation is maintained only in a CG dinucleotide (*black line*) in the Ubi-pro region. The results from at least 15 cloned sequences are shown

Although the non-coding Pol II transcript could still be detected in T + S + SD line #4 in *nrpd1* and *nrpe1* mutant backgrounds (Fig. 3c), CHG and CHH methylation at the 88 bp target region in both T + S + SD lines #1 and #4 was dramatically reduced in these mutants (Fig. 4). Because the Pol IV-dependent secondary siRNAs made in the T + S system are below detection levels in *nrpd1* and *nrpe1* mutants (Kanno et al. 2008; Daxinger et al. 2009), these results provide additional evidence that the secondary siRNAs acting in *trans* are involved in provoking methylation of the 88 bp target region.

**Fig. 5:**
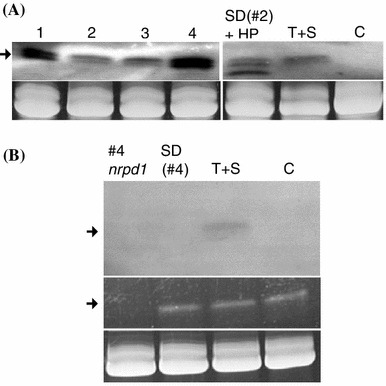
Northern blot analysis of siRNAs. **a** A probe specific for the 88 bp target sequence was used on Northern blots to detect siRNAs in T + S + SD lines #1 though #4 (*left blot*) as well as the original T + S line (T + S) and SD(#2) carrying the 88 bp-HP construct (SD(#2) + HP) (*right blot*). The *arrow* to the *left* indicates the position of the 24-nt size class. **B.** The 88 bp-specific probe was used to detect 24-nt siRNAs (*arrow*, *left*) in the original T + S line as well as T + S + SD line #4 in an *nrpd1* mutant background (#4-*nrpd1*) and line SD(#4). In **a** and **b**, the control *lane (C)* contains RNA isolated from non-transgenic plants. Ethidium bromide staining of the major RNA on the gel is shown at the *bottom* of each blot as a loading control. In **b**, the *middle panel* shows the 24-nt size class of siRNAs (*arrow*) on the stained gel. This size class disappears in the *nrpd1* mutant (lane #4 *nrpd1*) confirming the genotype of this plant

To analyze siRNAs, Northern blots were performed using an 88 bp-specific probe and RNA isolated from T + S + SD lines #1, #2, #3 and #4 (line #5 was not included in this analysis). Unexpectedly, elevated levels of 24-nt siRNAs were detected in the methylated T + S + SD lines #1 and #4 (Fig. 5a, lanes 1 and 4) whereas 24-nt siRNAs in the unmethylated lines #2 and #3 were present at roughly the same level as in the original T + S line (Fig. 5a, lanes 2, 3 and T + S). The similar abundance levels of 24-nt siRNAs in lines #2, #3 and T + S suggest that these siRNAs correspond to the original Pol IV-dependent secondary siRNAs made downstream of the target enhancer in the original T + S line (Fig. 1b). By contrast, the increased levels of 24-nt siRNAs observed in the lines #1 and #4 are suggestive of an amplification process occurring in the presence of the SD construct containing the 88 bp target sequence.

Because it was only observed in lines #1 and #4, siRNA amplification is likely to depend on certain features of the 88 bp target sequence—such as overlapping Pol II transcripts or DNA methylation—present specifically in these lines. Any involvement of the Pol II transcript in siRNA amplification, however, does not necessitate turnover of this RNA. This is inferred from the observation that the level of overlapping Pol II transcript was approximately the same in wild-type T + S + SD line #4 (Fig. 3b, short1, lane 4), in which siRNAs are amplified (Fig. 5a, lane 4), as in line #4 *nrpd1* mutant background (Fig. 3c, 4 *nrpd1*, ‘+’ lanes), which lacks detectable siRNAs hybridizing to the 88 bp-specific probe (Fig. 5b, lane 4-d1).

**Fig. 6:**
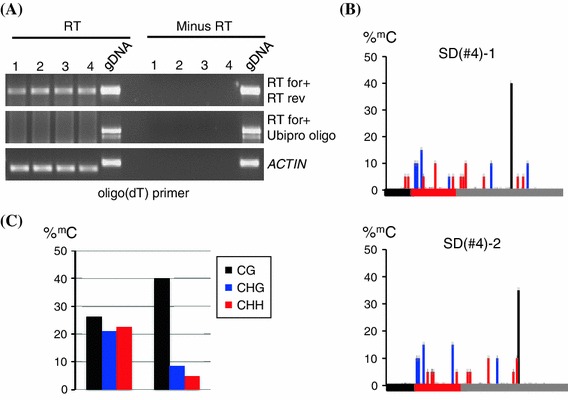
Analysis of Pol II transcript and methylation of 88 bp target sequence in line SD(#4). **a** RT-PCR analysis of the transcript overlapping the 88 bp target region in the line SD(#4). Four individual plants were tested. Positions of the primers used are shown in the Fig. 3a. An oligo(dT) primer was used for the RT reaction and primers for PCR were either RTfor + RTrev, which detects the ‘short’ transcript (Fig. 3a, b) or RTfor + Ubipro Oligo, which does not detect a transcript (Fig. 3b). ‘Minus RT’ indicates reactions without reverse transcriptase. *ACTIN* was used as a positive control for expression. gDNA, genomic DNA. **b** Percent cytosine methylation in the 88 bp target sequence (*red bar*) and immediate flanking sequences (*left black bar*, vector sequence; *right grey bar*, Ubi-pro sequence) in line SD (#4) as determined by bisulfite sequencing. Results from at least 15 cloned sequences from two individual plants are shown. CG, CHG and CHH are indicated by the *black*, *blue* and *red lines*, respectively. **c** The *graphs* show the comparison of overall levels of methylation in CG (*black*), CHG (*blue*) and CHH (*red*) nucleotide groups between line T + S + SD line #4 (*left*) and line D(#4) (*right*). Original bisulfite data for line T + S + SD line #4 are shown in Fig. 2b

Withdrawing the original source of *trans*-acting 24-nt secondary siRNAs by segregating the T and S loci away from the SD locus in line #4, producing line SD(#4), abolished the accumulation of detectable 24-nt siRNAs [Fig. 5b, lane SD (#4)] while not substantially affecting Pol II transcript levels (Fig. 6a, lanes 1–4). These results demonstrate that the *trans*-acting secondary siRNAs are needed continually for the siRNA amplification process and that the Pol II transcript alone is not sufficient to stimulate siRNA amplification. Moreover, in the absence of 24-nt siRNAs, the Pol II transcript alone is unable to promote efficient methylation at the 88 bp target sequence as indicated by the substantial reduction of CHG and CHH methylation observed in line SD(#4) (Fig. 6b, c). It is difficult to assess whether DNA methylation of the 88 bp target sequence has a role in siRNA amplification because mutations that disrupt RdDM also reduce methylation at the original T locus and hence abolish synthesis of secondary siRNAs, which are needed to trigger methylation of the 88 bp target region (Daxinger et al. 2009).

**Fig. 7:**
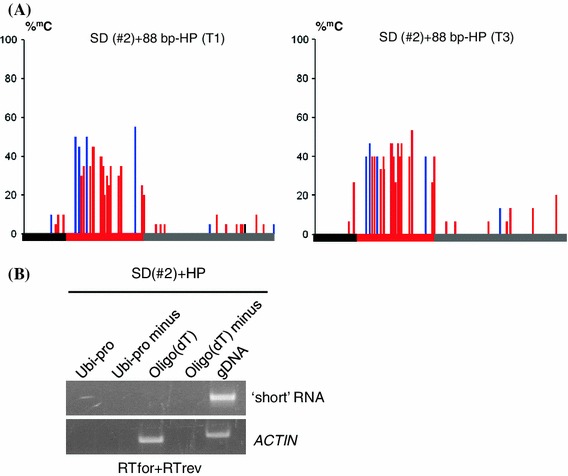
Methylation of 88 bp target sequence induced by 88 bp-HP construct and absence of Pol II transcript in line SD(#2). **a** Percent cytosine methylation in the 88 bp target sequence (*red bar*) and immediate flanking sequences (*left black bar*, vector sequence; *right grey bar*, Ubi-pro sequence) in line SD(#2) in T1 and T3 generations when the 88 bp-HP locus is hemizygous and homozygous, respectively. CG, CHG and CHH are indicated by the *black*, *blue* and *red lines*, respectively. The results from at least 15 cloned sequences are shown. **b** RT-PCR analysis of the transcript overlapping the 88 bp target region in line SD(#2). Either a Ubi-pro or oligo(dT) primer was used for the RT reaction (*top*) and primers for PCR were RTfor + RTrev, which detects the ‘short’ transcript (Fig. 3a, b). ‘Minus’ *lanes 2* and *4* indicate reactions without reverse transcriptase. *ACTIN* was used as a positive control for expression. gDNA, genomic DNA

As described above, Pol IV-dependent *trans*-acting secondary siRNAs failed to induce methylation of the 88 bp target sequence in the T + S + SD lines #2 and #3, which also lacked the overlapping Pol II non-coding transcript. To determine whether the 88 bp target sequence in these lines is completely resistant to RdDM or just insensitive to methylation induced by *trans*-acting secondary siRNAs, a different strategy for producing siRNAs was tested. After segregating away the T and S loci from the SD locus in line #2 by repeated backcrossing to non-transgenic plants, producing line SD(#2), a transgene construct containing an inverted DNA repeat of the 88 bp sequence under the control of the cauliflower mosaic virus 35S promoter (88 bp-HP) was introduced. Transcription of this construct by Pol II is predicted to produce a hairpin RNA that is processed redundantly by DCL4, DCL2 and DCL3 to generate 21-, 22-, and 24-nt siRNAs, respectively (Dunoyer et al. 2007; Daxinger et al. 2009). In the presence of the 88 bp-HP construct, the 88 bp target sequence in line SD(#2) acquired persistent methylation (Fig. 7a), and as expected, hairpin-derived siRNAs 21–24-nt in length could be detected in methylated plants (Fig. 5a, lane SD(#2) +HP). These results demonstrate that the 88 bp target sequence in line #2 is not recalcitrant to RdDM but perhaps requires siRNAs that have specific features, for example certain lengths or abundance levels. The findings also indicate that an overlapping Pol II-generated transcript, which was not detected in T + S + SD line #2 (Fig. 3b, lane 2, short 1 and 2) or in line SD(#2) containing the 88 bp-HP construct (Fig. 7b), is not required for methylation triggered by hairpin-derived siRNAs.

The results of all experiments are summarized in Table 1.

## Discussion

A number of studies have shown that 21–24-nt hairpin-derived siRNAs, which depend on Pol II transcription of inverted DNA repeats, can act in *trans* to trigger DNA methylation of unlinked homologous target sequences (Kanno et al. 2005, 2008; Eamens et al. 2008; Finke et al. 2012; this study). It has not been clear, however, whether Pol IV/RDR2-dependent 24-nt siRNAs, which can induce methylation in *cis* at the site where they are generated (Lister et al. 2008; Daxinger et al. 2009), can similarly elicit RdDM in *trans*. Here we show that Pol IV/RDR2-dependent, 24-nt secondary siRNAs are able to act in *trans* to induce DNA methylation of an unlinked homologous target sequence, provided this sequence is transcribed by Pol II to produce a non-coding RNA. As discussed below, both the Pol II transcript and Pol IV-dependent, *trans*-acting secondary siRNAs appear to be required for amplification of 24-nt siRNAs at the unlinked target site, presumably to achieve a locally high level that is sufficient to induce RdDM through Pol V pathway components.

**Fig. 8:**
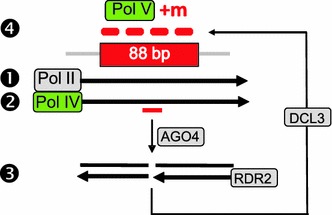
Model for roles of RNA polymerases II, IV and V in RdDM at the 88 bp target sequence. *Step 1* Initiating at an unidentified promoter in flanking plant DNA, Pol II transcribes a non-coding RNA that overlaps the 88 bp target region and terminates in the Ubi-pro. *Step 2* Pol II transcription (or transcripts) recruits Pol IV to transcribe through the 88 bp target region. *Step 3* *Trans*-acting secondary siRNAs (*short red bar*) matching the 88 bp target sequence may guide AGO4 cleavage of the ‘aberrant’ Pol IV transcript, thus initiating siRNA amplification at the 88 bp target region by providing substrates for RDR2, which produces double stranded RNA that is processed by DCL3 to 24-nt siRNAs. *Step 4* The amplified siRNAs (*thick red bars*) reach a sufficiently high local concentration to induce Pol V-mediated methylation (*red* ‘+m’) of the 88 bp target sequence. The results leading to this model provide experimental validation of the RNA silencing cascade model proposed previously (Baulcombe 2006)

Three types of RNA have a role in RdDM of the 88 bp target region at the SD locus in our system (Table 1): (1) an overlapping Pol II-generated non-coding transcript, which apparently initiates at a promoter in flanking plant DNA; (2) Pol IV-dependent, *trans*-acting 24-nt secondary siRNAs produced at the T locus in the original T + S line; and (3) amplified 24-nt siRNAs, which are presumably generated by Pol IV pathway components at the unlinked 88 bp target region. Although the precise roles of these three RNA species in RdDM of the 88 bp target sequence are not yet fully understood, the results can be interpreted in the context of a model (Fig. 8) that draws on a previous proposal that Pol II transcription (or transcripts) can recruit Pol IV and Pol V to chromatin to act in siRNA biogenesis and DNA methylation, respectively (Zheng et al. 2009).

The evidence suggesting that Pol II may recruit Pol V in our system is that *trans*-acting secondary siRNAs fail to induce RdDM of the 88 bp target sequence in T + S + SD lines #2 and #3 that lack a Pol II transcript overlapping this sequence (Table 1). However, an alternate explanation for this finding is that the unamplified *trans*-acting secondary siRNAs are not abundant enough to induce methylation of the 88 bp sequence on their own. Therefore, our data are suggestive but not conclusive on the necessity of Pol II to directly recruit Pol V to orchestrate RdDM of the 88 bp target sequence. Nevertheless, the results indicate that unamplified *trans*-acting siRNAs alone are not sufficient to induce RdDM of the 88 bp target sequence. Moreover, the converse is also true: the Pol II transcript alone is unable to support full methylation in the absence of 24-nt siRNAs (Table 1).

The evidence for recruitment of Pol IV by Pol II in our system is that similarly to DNA methylation, siRNA amplification—which is presumed to require Pol IV (Fig. 8)—also does not occur in lines #2 and #3 that lack a Pol II transcript (Table 1). Pol II is envisioned to have an indirect role in siRNA amplification through its previously documented ability to recruit Pol IV (Zheng et al. 2009). The proposed indirect role of Pol II in siRNA amplification is supported by the observation that the Pol II transcript accumulates to similar levels whether siRNAs are amplified or not (Table 1). The relative stability of the Pol II transcript contrasts to the presumed turnover of a putative Pol IV transcript when secondary siRNAs are generated in the T + S system (Daxinger et al. 2009).

In addition to a requirement for the overlapping Pol II transcript, siRNA amplification also depends on *trans*-acting secondary siRNAs because amplification does not occur in plants that contain the Pol II transcript but lack the secondary siRNAs (Table 1). There are at least two ways that the *trans*-acting secondary siRNAs could function in siRNA amplification, which is proposed to take place at the unlinked 88 bp target site following Pol II-dependent recruitment of Pol IV (Fig. 8). First, they may induce a low level of methylation at the 88 bp sequence that assists in attracting Pol IV to the target region. Second, they may guide AGO4 cleavage of a Pol IV transcript to provide substrates for RDR2 in the siRNA amplification pathway (Fig. 8).

In our model, the *trans*-acting secondary siRNAs and overlapping Pol II transcript are proposed to be involved primarily in siRNA amplification at the 88 bp target site. By contrast, the amplified 24-nt siRNAs themselves are likely to play the critical role in triggering DNA methylation in *cis* of the 88 bp sequence. Their ability to do so may depend on their higher abundance relative to the unamplified *trans*-acting secondary siRNAs and on their proximity to the 88 bp target locus. If Pol II transcripts (or transcription) are indeed able to recruit Pol IV to the 88 bp target region as suggested above, then following Pol IV-dependent amplification, the resulting amplified siRNAs would be locally available at a relatively high concentration to guide DNA methylation at the 88 bp sequence (Fig. 8).

It is interesting that the 88 bp target sequence in T + S + SD line #2 that did not acquire methylation in the presence of *trans*-acting, 24-nt secondary siRNAs nevertheless became methylated in the presence of 21–24-nt hairpin-derived siRNAs, even in the absence of an overlapping Pol II transcript. This result indicates that the 88 bp target sequence in line #2 is not inherently resistant to siRNA-mediated methylation and that an overlapping Pol II transcript is not essential for RdDM at this sequence. The hairpin-derived siRNAs do not appear more abundant than *trans*-acting secondary siRNAs on the Northern blots, but we cannot rule out that abundance levels or availability of siRNAs are important in determining whether somewhat resistant targets, such as the 88 bp sequence in line #2, become methylated. A second consideration is that the hairpin-derived siRNAs comprise a heterogeneous population resulting from the redundant action of several different DCL enzymes. Although the identification of *dcl3* mutants in forward genetic screens has suggested that 24-nt siRNAs are most effective in inducing RdDM (Daxinger et al. 2009; Greenberg et al. 2011), recent work has demonstrated that 21-nt siRNAs are important for methylation at some loci (Pontier et al. 2012; Wu et al. 2012). Therefore, it is conceivable that hairpin-derived 21-nt siRNAs act together with 24-nt siRNAs to enhance the efficiency of RdDM at the non-transcribed 88 bp target sequence in line SD(#2).

Our study may have uncovered a role for Pol II in RdDM because the 88 bp target sequence, in the context of the SD construct, resembles to some extent the low copy, intergenic (Type II) loci that were previously shown to require Pol II transcripts or transcription for effective TGS mediated by Pol IV and Pol V (Zheng et al. 2009). It remains unclear why at least some Type II loci require Pol II to coordinate the activities of Pol IV and Pol V. Perhaps a non-coding Pol II transcript provides some kind of a signal at the chromatin or RNA level that recruits Pol IV, which in turn produces non-polyadenylated, non-coding transcripts that are perceived as ‘aberrant’ and enter into the siRNA biogenesis pathway (Fig. 8). In *Arabidopsis*, 3.6 % of the intergenic space is transcribed by Pol II to produce polyadenylated transcripts, of which only about one-third may be translated into proteins (Moghe et al. 2013). Conceivably, some of the non-coding, intergenic Pol II transcripts may be involved in coordinating Pol IV and Pol V activities to act in siRNA-mediated amplification and RdDM, respectively. Pol II transcripts of a novel class of microRNA genes have also been suggested to be involved in biogenesis of Pol IV/RDR2-dependent siRNAs that direct AGO4-dependent methylation of target genes in *trans* (Chellappan et al. 2010).

The ability of Pol IV/RDR2-dependent siRNAs to induce methylation in *trans* may be important for establishing global silencing of transposable element (TE) families. If a single (perhaps rearranged) copy of a TE generates Pol IV/RDR2-dependent siRNAs that act in *trans* to amplify siRNAs at Pol II-transcribed copies dispersed throughout the genome, eventually all TE family members, even those not originally recognized by Pol IV/Pol V, could potentially become silenced. Although the 88 bp target sequence studied here is more than twice the length of the minimum target size required for RdDM (which is approximately 30 bp; Pélissier and Wassenegger 2000), longer sequences or sequences with a higher content of symmetrical CG and CHG nucleotide groups, which efficiently maintain methylation (Meyer 2011), may be even more susceptible to *trans*-RdDM.

## Electronic supplementary material

Below is the link to the electronic supplementary material.

Supplementary material 1 (DOC 53 kb)

Supplementary material 2 (PDF 152 kb)
