# Supplementary material for: Interplay among RNA polymerases II, IV and V in RNA-directed DNA methylation at a low copy transgene locus in Arabidopsis thaliana
Source: Plant Mol Biol. 2013 Mar 20;82(1):85–96. doi: 10.1007/s11103-013-0041-4 (PMC3646161; doi:10.1007/s11103-013-0041-4)
Supplement: Supplementary file 1 — Supplementary material 1 (DOC 53 kb) [file 11103_2013_41_MOESM1_ESM.doc]

**Supplementary Table1. Primers**

**Primers used for genotyping**

| **primer** | **sequence** |
| --- | --- |
| *nrpd1-7* *BamH*I forward | 5’-CCT TTT TGC AGG TTT ATG CTC-3’ |
| *nrpd1-7* *BamH*I reverse | 5’-GTT TCT TGT AGA TCC GAG TCC GTG GAT C-3’ |
| *nrpe1-3* *Dra*I forward | 5’-ACT GGA GAT GCT TAC CGA CAT GTG AAT GA-3’ |
| *nrpe1-3* *Dra*I reverse | 5’-GGT AGA ACA AAT GGA CAC AAT CAC CTT-3’ |
| Target co-dominant forward | 5’-GGA CAC ATC CTA TAG TTC GC-3’ |
| Target co-dominant reverse | 5’-GAT CTA CCC ACT AAT CTA CTC G-3’ |
| Target co-dominant internal | 5’-GGC GAA CCA AGC CGC TAA TGC-3’ |
| Silencer co-dominant forward | 5’-GGT CAT CAC AAA CAT CTC GT-3’ |
| Silencer co-dominant reverse | 5’-GTG ATC CAA AGT CAT GGT CT-3’ |
| Silencer co-dominant internal | 5’-CAG CCG TCC AAA TGC GGG AT-3’ |

**Primers used for bisulfite sequencing**

| **primer** | **sequence** |
| --- | --- |
| Phavoluta primary forward | 5’-GTG YAG ATY TGT TTG GAG YTG ATT Y-3’ |
| Phavoluta primary reverse | 5’-TTT AAT ATC TAA CAT AAC CAA CCT TT-3’ |
| Phavoluta secondary forward | 5’-GGA YYA TAG TGA TGY YAT ATT GTG-3’ |
| Phavoluta secondary reverse | 5’-TAT CAT CAA CAA CTT TCC ACA CC-3’ |
| 88bp-ubi-dsred forward | 5’-TGA TAG TTT AAA TTG AAG GYG GGA AAY GAT AAT-3’ |
| 88bp-ubi-dsred reverse | 5’-ACT CAA TTA TCC TTT AAA CCA TAT CTA ACT ATT C-3’ |
| Target forward | 5’-GCG GTG TYA TYT ATG TTA YTA GAT-3’ |
| Target reverse | 5’-CTT CTT RAT RTT CCA TAR CTT TCC-3’ |

**Primers used for RT-PCR and Pol II ChIP**

| **Primer** | **Sequence** |
| --- | --- |
| Ubi ChIP short for | 5’- TTG ACA ACA GGA CTC TAC AG-3’ |
| Ubi ChIP short rev | 5’-AAA CCA TTA ACC CTA AAC C-3’ |
| Ubi ChIP long for | 5’- CAC CAA CCA GCG AAC CAG CA-3’ |
| Ubi ChIP long rev | 5’-ATT TCT GGA TGC CGA CAG-3’ |
| *Actin* for ChIP forward | 5’-CGT TTC GCT TTC CTT AGT GTT AGC T-3’ |
| *Actin* for ChIP reverse | 5’-AGC GAA CGG ATC TAG AGA CTC ACC TTG-3’ |
| *IGN5* for ChIP forward | 5’-TCC CGA GAA GAG TAG AAC AAA TGC TAA AA-3’ |
| *IGN5* for ChIP reverse | 5’-CTG AGG TAT TCC ATA GCC CCT GAT CC-3’ |
| dsRED for | 5’-ATG GAC AAC ACC GAG GAC GT-3’ |
| dsRED rev | 5’- CTA CTG GGA GCC GGA GTG GC-3’ |
| RT for | 5’-CGA CAA TCT GAT CCA CTA-3’ |
| RT rev | 5’-AAA CCA TTA ACC CTA AAC C-3’ |
| Ubipro oligo | 5’-ATT TCT GGA TGC CGA CAG-3’ |
| At3g18780 (*act2*) forward | 5’-GCC ATC CAA GCT GTT CTC TC-3’ |
| At3g18780 (*act2*) reverse | 5’-GGG CAT CTG AAT CTC TCA GC-3’ |
