# Supplementary material for: Interplay among RNA polymerases II, IV and V in RNA-directed DNA methylation at a low copy transgene locus in Arabidopsis thaliana
Source: Plant Mol Biol. 2013 Mar 20;82(1):85–96. doi: 10.1007/s11103-013-0041-4 (PMC3646161; doi:10.1007/s11103-013-0041-4)
Supplement: Supplementary file 2 — Supplementary material 2 (PDF 152 kb) [file 11103_2013_41_MOESM2_ESM.pdf]

```
                                         RT for
1     TCAAACACTG ATAGTTTAAA CTGAAGGCGG GAAACGACAA TCTGATCCAC TAGTTCTAGT AATAATAAAC AGGCTGCATC TTCAGGCATC CAAAAATTCC
      AGTTTGTGAC TATCAAATTT GACTTCCGCC CTTTGCTGTT AGACTAGGTG ATCAAGATCA TTATTATTTG TCCGACGTAG AAGTCCGTAG GTTTTTAAGG

101   TCTCTTCTCT CTAAAGATAC TTCTTAAAGA TAAAAATTCA GGAAAGCTCT AGAGATAATG AGCATTGCAT GTCTAAGTTA TAAAAAATTA CCACATATTT
      AGAGAAGAGA GATTTCTATG AAGAATTTCT ATTTTTAAGT CCTTTCGAGA TCTCTATTAC TCGTAACGTA CAGATTCAAT ATTTTTTAAT GGTGTATAAA

201   TTTTTGTCAC ACTTGTTTGA AGTGCAGTTT ATCTATCTTT ATACATATAT TTAAACTTTA CTCTACGAAT AATATAATCT ATAGTACTAC AATAATATCA
      AAAAACAGTG TGAACAAACT TCACGTCAAA TAGATAGAAA TATGTATATA AATTTGAAAT GAGATGCTTA TTATATTAGA TATCATGATG TTATTATAGT

                                                                  Ubi ChIP short for
301   GTGTTTTAGA GAATCATATA AATGAACAGT TAGACATGGT CTAAAGGACA ATTGAGTATT TTGACAACAG GACTCTACAG TTTTATCTTT TTAGTGTGCA
      CACAAAATCT CTTAGTATAT TTACTTGTCA ATCTGTACCA GATTTCCTGT TAACTCATAA AACTGTTGTC CTGAGATGTC AAAATAGAAA AATCACACGT

401   TGTGTTCTCC TTTTTTTTTG CAAATAGCTT CACCTATATA ATACTTCATC CATTTTATTA GTACATCCAT TTAGGGTTTA  GGGTTAATGG TTTTTATAGA
      ACACAAGAGG AAAAAAAAAC GTTTATCGAA GTGGATATAT TATGAAGTAG GTAAAATAAT CATGTAGGTA AATCCCAAAT CCCAATTACC AAAAATATCT
                                                                            RT rev/Ubi ChIP short rev
501   CTAATTTTTT TAGTACATCT ATTTTATTCT ATTTTAGCCT CTAAATTAAG AAAACTAAAA CTCTATTTTA GTTTTTTTAT TTAATAATTT AGATATAAAA
      GATTAAAAAA ATCATGTAGA TAAAATAAGA TAAAATCGGA GATTTAATTC TTTTGATTTT GAGATAAAAT CAAAAAAATA AATTATTAAA TCTATATTTT

601   TAGAATAAAA TAAAGTGACT AAAAATTAAA CAAATACCCT TTAAGAAATT AAAAAAACTA AGGAAACATT TTTCTTGTTT CGAGTAGATA ATGCCAGCCT
      ATCTTATTTT ATTTCACTGA TTTTTAATTT GTTTATGGGA AATTCTTTAA TTTTTTTGAT TCCTTTGTAA AAAGAACAAA GCTCATCTAT TACGGTCGGA
                                  Ubi ChIP long for
701   GTTAAACGCC GTCGACGAGT CTAACGGACA CCAACCAGCG AACCAGCAGC GTCGCGTCGG GCCAAGCGAA GCAGACGGCA CGGCATCTCT GTCGCTGCCT
      CAATTTGCGG CAGCTGCTCA GATTGCCTGT GGTTGGTCGC TTGGTCGTCG CAGCGCAGCC CGGTTCGCTT CGTCTGCCGT GCCGTAGAGA CAGCGACGGA

801   CTGGACCCCT CTCGAGAGTT CCGCTCCACC GTTGGACTTG CTCCGCTGTC GGCATCCAGA  AATTGCGTGG CGGAGCGGCA GACGTGAGCC GGCACGGCAG
      GACCTGGGGA GAGCTCTCAA GGCGAGGTGG CAACCTGAAC GAGGCGACAG CCGTAGGTCT TTAACGCACC GCCTCGCCGT CTGCACTCGG CCGTGCCGTC
                                             Ubipro oligo/Ubi ChIP long rev
901   GCGGCCTCCT CCTCCTCTCA CGGCACCGGC AGCTACGGGG GATTCCTTTC CCACCGCTCC TTCGCTTTCC CTTCCTCGCC CGCCGTAATA AATAGACACC
      CGCCGGAGGA GGAGGAGAGT GCCGTGGCCG TCGATGCCCC CTAAGGAAAG GGTGGCGAGG AAGCGAAAGG GAAGGAGCGG GCGGCATTAT TTATCTGTGG

1001  CCCTCCACAC CCTCTTTCCC CAACCTCGTG TTGTTCGGAG CGCACACACA CACAACCAGA TCTCCCCCAA ATCCACCCGT CGGCACCTCC GCTTCAAGgt
```

GGGAGGTGTG GGAGAAAGGG GTTGGAGCAC AACAAGCCTC GCGTGTGTGT GTGTTGGTCT AGAGGGGGTT TAGGTGGGCA GCCGTGGAGG CGAAGTTCca

1101 acgccgctcg tcctcccccc cccccctct ctaccttctc tagatcggcg ttccggtcca tggttagggc ccggtagttc tacttctgtt catgtttgtg
tgcggcgagc aggaggggg ggggggaga gatggaagag atctagccgc aaggccaggt accaatcccg ggccatcaag atgaagacaa gtacaaacac

1201 ttagatccgt gtttgtgtta gatccgtgct gctagcgttc gtacacggat gcgacctgta cgtcagacac gttctgattg ctaacttgcc agtgtttctc
aatctaggca caaacacaat ctaggcacga cgatcgcaag catgtgccta cgctggacat gcagtctgtg caagactaac gattgaacgg tcacaaagag

1301 tttggggaat cctgggatgg ctctagccgt tccgcagacg ggatcgattt catgattttt tttgtttcgt tgcatagggt ttggtttgcc cttttccttt
aaaccccctta ggaccctacc gagatcggca aggcgtctgc cctagctaaa gtactaaaaa aaacaaagca acgtatccca aaccaaacgg gaaaaggaaa

1401 atttcaatat atgccgtgca cttgtttgtc gggtcatctt ttcatgcttt tttttgtctt ggttgtgatg atgtggtctg gttgggcggt cgttctagat
taaagttata tacggcacgt gaacaaacag cccagtagaa aagtacgaaa aaaaacagaa ccaacactac tacaccagac caacccgcca gcaagatcta

1501 cggagtagaa ttaattctgt ttcaaactac ctggtggatt tattaatttt ggatctgtat gtgtgtgcca tacatattca tagttacgaa ttgaagatga
gcctcatctt aattaagaca aagtttgatg gaccacctaa ataattaaaa cctagacata cacacacggt atgtataagt atcaatgctt aacttctact

1601 tggatggaaa tatcgatcta ggataggtat acatgttgat gcgggtttta ctgatgcata tacagagatg ctttttgttc gcttggttgt gatgatgtgg
acctaccttt atagctagat cctatccata tgtacaacta cgcccaaaat gactacgtat atgtctctac gaaaaacaag cgaaccaaca ctactacacc

1701 tgtggttggg cggtcgttca ttcgttctag atcggagtag aatactgttt caaactacct ggtgtattta ttaattttgg aactgtatgt gtgtgtcata
acaccaaccc gccagcaagt aagcaagatc tagcctcatc ttatgacaaa gtttgatgga ccacataaat aattaaaacc ttgacataca cacacagtat

1801 catcttcata gttacgagtt taagatggat ggaaatatcg atctaggata ggtatacatg ttgatgtggg ttttactgat gcatatacat gatggcatat
gtagaagtat caatgctcaa attctaccta cctttatagc tagatcctat ccatatgtac aactacaccc aaaatgacta cgtatatgta ctaccgtata

1901 gcagcatcta ttcatatgct ctaaccttga gtacctatct attataataa acaagtatgt tttataatta ttttgatctt gatatacttg gatgatggca
cgtcgtagat aagtatacga gattggaact catggataga taatattatt tgttcataca aaatattaat aaaactagaa ctatatgaac ctactaccgt

2001 tatgcagcag ctatatgtgg attttttag ccctgccttc atacgctatt tatttgcttg gtactgtttc ttttgtcgat gctcaccctg ttgtttggtg
atacgtcgtc gatatacacc taaaaaaatc gggacggaag tatgcgataa ataaacgaac catgacaaag aaaacagcta cgagtgggac aacaaaccac

```
                                                                dsRED for
2101   ttacttctgc agGTCGACTC TAGAGATATC AAGCTTGGGG TACCGGTCGC CACCATGGAC AACACCGAGG ACGTCATCAA GGAGTTCATG CAGTTCAAGG
       aatgaagacg tcCAGCTGAG ATCTCTATAG TTCGAACCCC ATGGCCAGCG GTGGTACCTG TTGTGGCTCC TGCAGTAGTT CCTCAAGTAC GTCAAGTTCC

2201   TGCGCATGGA GGGCTCCGTG AACGGCCACT ACTTCGAGAT CGAGGGCGAG GGCGAGGGCA AGCCCTACGA GGGCACCCAG ACCGCCAAGC TGCAGGTGAC
       ACGCGTACCT CCCGAGGCAC TTGCCGGTGA TGAAGCTCTA GCTCCCGCTC CCGCTCCCGT TCGGGATGCT CCCGTGGGTC TGGCGGTTCG ACGTCCACTG

2301   CAAGGGCGGC CCCCTGCCCT TCGCCTGGGA CATCCTGTCC CCCCAGTTCC AGTACGGCTC CAAGGCCTAC GTGAAGCACC CCGCCGACAT CCCCGACTAC
       GTTCCCGCCG GGGGACGGGA AGCGGACCCT GTAGGACAGG GGGGTCAAGG TCATGCCGAG GTTCCGGATG CACTTCGTGG GGCGGCTGTA GGGGCTGATG

2401   ATGAAGCTGT CCTTCCCCGA GGGCTTCACC TGGGAGCGCT CCATGAACTT CGAGGACGGC GGCGTGGTGG AGGTGCAGCA GGACTCCTCC CTGCAGGACG
       TACTTCGACA GGAAGGGGCT CCCGAAGTGG ACCCTCGCGA GGTACTTGAA GCTCCTGCCG CCGCACCACC TCCACGTCGT CCTGAGGAGG GACGTCCTGC

2501   GCACCTTCAT CTACAAGGTG AAGTTCAAGG GCGTGAACTT CCCCGCCGAC GGCCCCGTAA TGCAGAAGAA GACTGCCGGC TGGGAGCCCT CCACCGAGAA
       CGTGGAAGTA GATGTTCCAC TTCAAGTTCC CGCACTTGAA GGGGCGGCTG CCGGGGCATT ACGTCTTCTT CTGACGGCCG ACCCTCGGGA GGTGGCTCTT

2601   GCTGTACCCC CAGGACGGCG TGCTGAAGGG CGAGATCTCC CACGCCCTGA AGCTGAAGGA CGGCGGCCAC TACACCTGCG ACTTCAAGAC CGTGTACAAG
       CGACATGGGG GTCCTGCCGC ACGACTTCCC GCTCTAGAGG GTGCGGGACT TCGACTTCCT GCCGCCGGTG ATGTGGACGC TGAAGTTCTG GCACATGTTC

2701   GCCAAGAAGC CCGTGCAGCT GCCCGGCAAC CACTACGTGG ACTCCAAGCT GGACATCACC AACCACAACG AGGACTACAC CGTGGTGGAG CAGTACGAGC
       CGGTTCTTCG GGCACGTCGA CGGGCCGTTG GTGATGCACC TGAGGTTCGA CCTGTAGTGG TTGGTGTTGC TCCTGATGTG GCACCACCTC GTCATGCTCG

2801   ACGCCGAGGC CCGCCACTCC GGCTCCCAGT AG
       TGCGGCTCCG GGCGGTGAGG CCGAGGGTCA TC
                         dsRED rev
```

Supplementary Figure 1: Sequence of the SD construct.

The figure represents the DNA sequence of the SD construct (2,832 bp) containing the 88 bp target region (red letters), maize ubiquitin promoter (extending from 158 bp to 2,112 bp; large intron in lower case letters) (Christensen et al., 1992), and the gene encoding the DsRed protein (2,155 bp to the end). The part of the 88 bp sequence boxed in black (nt 70-91) is included in the probe used in Northern blot analyses to detect both secondary siRNAs and hairpin-derived siRNAs. The probe itself is somewhat longer at the 5' end (Methods section) but owing to the presence of an ATG, this extension was not included in the 88 bp target sequence to avoid short open reading frames in the target sequence. The five sequences boxed in blue indicate the primers used to detect the noncoding RNA in RT-PCR experiments and ChIP analysis to assess Pol II occupancy (Fig. 3A-D). The solid underline (ca. 35 bp to 870 bp) indicates the extent of the non-coding RNA as predicted from the RT-PCR results (Fig. 3A, B). Uncertainty in the exact 5' and 3' ends is denoted by the dotted underline. Regions used to design degenerate primers for bisulfite sequencing (Suppl. Table 1) to detect methylation in the 88 bp target region and start of the Ubi-pro are shown in pink letters. The putative TATA box in the ubiquitin promoter is shown in green shaded letters and the transcription start site is shaded in blue. The start codon ATG site (nt 2155) and stop codon TAG site (nt 2830) of the *DsRed* gene are shaded in black.

Christensen AH, Sharrock RA, Quail PH (1992) Maize polyubiquitin genes: structure, thermal perturbation of expression and transcript splicing, and promoter activity following transfer to protoplasts by electroporation. Plant Mol Biol 18: 675-689.
